# The prognostic value of osteopontin in limited-stage small cell lung cancer patients and its mechanism

**DOI:** 10.18632/oncotarget.19589

**Published:** 2017-07-26

**Authors:** Fanglei Liu, Chunxue Bai, Zhongliang Guo

**Affiliations:** ^1^ Department of Respiratory Medicine, Zhongshan Hospital Affiliated to Fudan University, Shanghai, China; ^2^ Department of Respiratory Medicine, Dongfang Hospital Affiliated to Tongji University, Shanghai, China

**Keywords:** small cell lung cancer, osteopontin, prognosis, proliferation, epithelial-mesenchymal transition

## Abstract

Osteopontin (OPN) is known to be overexpressed in numerous carcinomas. Although abundant OPN has been reported to be correlated with poor survival in non-small cell lung cancer (NSCLC) but their clinical and prognostic significance in SCLC remains unknown. In this study, RNA-sequencing was used to obtain gene expression data in SCLC tissue samples and OPN expression levels were then investigated using qPCR, immunohistochemical and Western blot. We found OPN was one of the most upregulated genes. Besides, the correlation of OPN with tumor clinicopathological characteristics was evaluated and we found OPN was associated with advanced tumor stages. In addition, Kaplan-Meier survival analysis and Cox analyses revealed OPN expression was an independent predictor for overall survival (OS) (P= 0.013) and progression-free survival (PFS) (P=0.008). A high level of OPN was correlated with pT classification and pN classification (P<0.05). Moreover, *In vitro* experiments, by test the biological function of OPN via colony formation, wound healing, Transwell assays, and western blotting, we found that overexpression of OPN induced cell proliferation, migration, and invasion; down-regulation of OPN inhibited these. Overexpression of OPN stimulates epithelial-mesenchymal transition (EMT) whereas OPN silencing prevents the EMT. In conclusion: OPN appears to contribute to the malignant mechanism of SCLC and is a promising and significant prognostic predictor in patients with SCLC. Specific silence of OPN could be a future direction to develop a novel therapeutic strategy for SCLC patients.

## INTRODUCTION

Lung cancer accounts for 12% of all new cases of cancers worldwide, it is the second most common cancer in men and women. Small cell lung cancer (SCLC) accounts for about 15% of primary lung cancers, and shows a rapid growth rate and early development of widespread metastases compared to NSCLC [1]. Chemotherapy is the main therapy method for SCLC in combined treatment. Although SCLC is sensitive to chemotherapy in the early stages of therapy, SCLC often recurs with multi-drug resistance [2]. Thus, the prognosis of SCLC is very poor, and the overall 5-year survival rate is only 5% [3]. Improving the understanding of the pathogenic factors involved in the progression of SCLC and developing biomarkers for prognosis are important for improving diagnostic and therapeutic procedures.

Osteopontin (OPN) is a multifunctional secreted phosphorylated glycoprotein involved in the regulation of cell adhesion, migration, and invasion via binding to integrins and CD44 receptors [4, 5]. Elevated OPN levels have been reported in various malignant tumors, including breast, liver, gastric, NSCLC, and others [6]. Researchers have demonstrated that OPN over-expression is strongly associated with tumor progression and poor prognosis [4, 7, 8]. In SCLC, OPN reduces cisplatin-induced cell apoptosis and induces chemotherapy resistance [9]. In addition, researchers suggested that tumor-derived OPN stimulates the cancer-associated fibroblast (CAF) phenotype in mesenchymal stem cells [10], which participate in tumor progression by secreting several growth factors, stimulating angiogenesis, and inducing epithelial-mesenchymal transition (EMT). Accumulating evidence suggests that high OPN expression is associated with a poor clinical prognosis in various cancers, including pancreas, colon, breast cancers and NSCLC [11-14]. Despite numerous studies indicated the prognostic significance of OPN in a variety of carcinomas, in SCLC, the relationship between OPN expression and clinico-pathological features remains unclear.

So far, no researches used RNA-sequencing technic to investigated gene expression data in SCLC. Here, we identified 96 significant aberrant mRNA and OPN was one of most upregulated genes. Based on previous studies, we hypothesized that OPN expression in SCLC may be an independent predictor of malignant behavior. To evaluate the clinical significance of OPN in SCLC, we further examined its expression in the tissues of patients with SCLC through integrated approaches and compared the results with clinicopathological findings, and *in vitro* biological experiments were performed to investigate the potential mechanism.

## RESULTS

### OPN expression is frequently upregulated in SCLC

**Figure 1:**
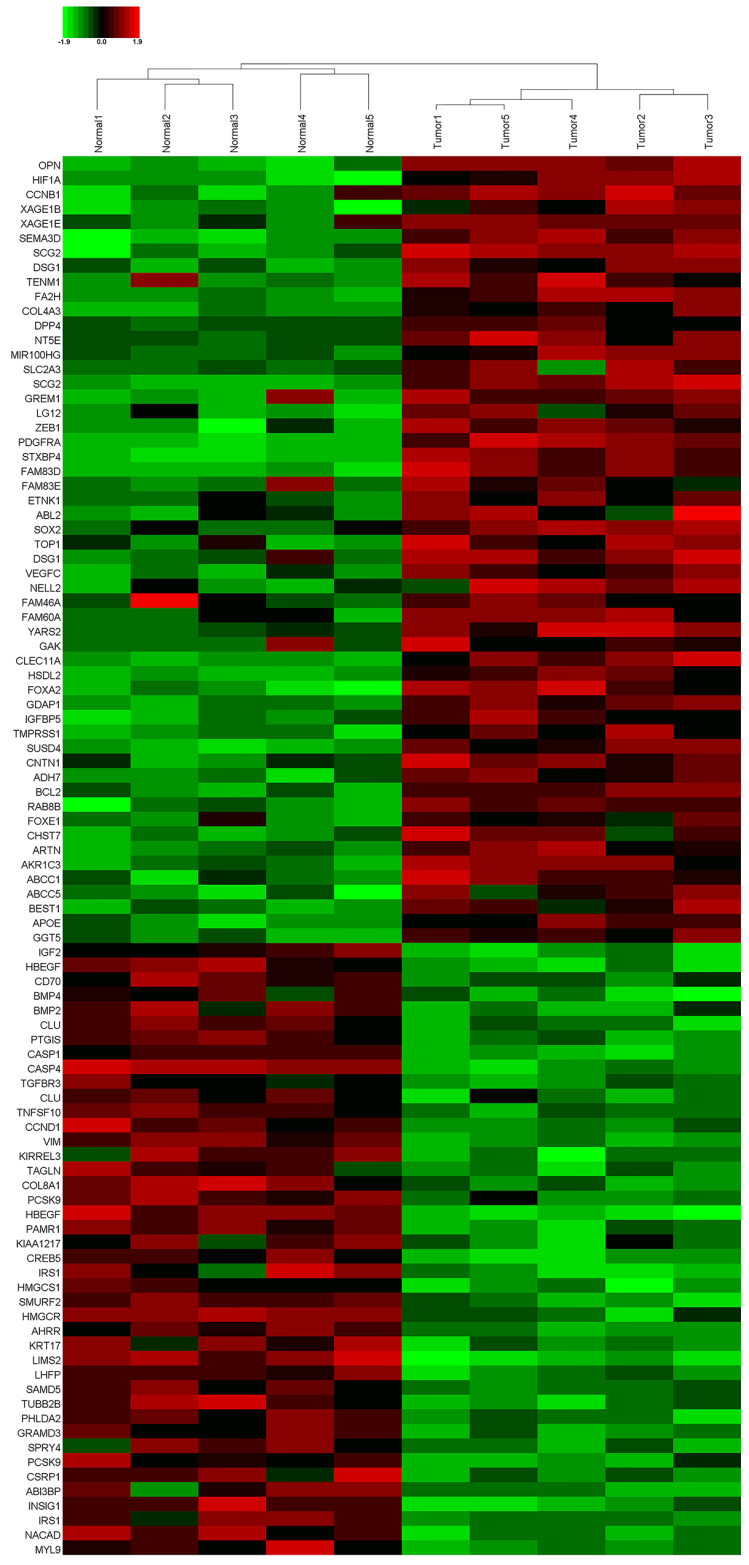
A cluster diagram of RNA-sequencing data from five pairs of small cell lung cancer (SCLC) tissues samples The green bar means downregulated, red part means upregulated.

**Figure 2:**
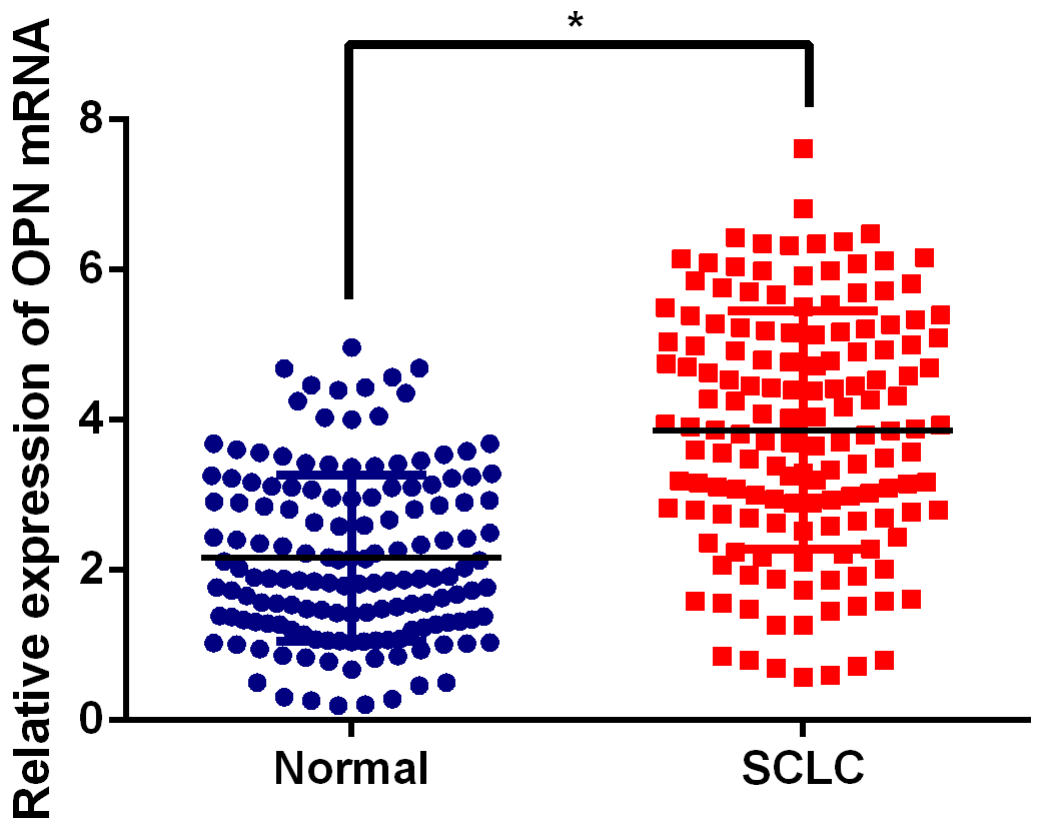
Expression levels of OPN in SCLC tissues and paired normal tissue samples by qPCR

**Figure 3:**
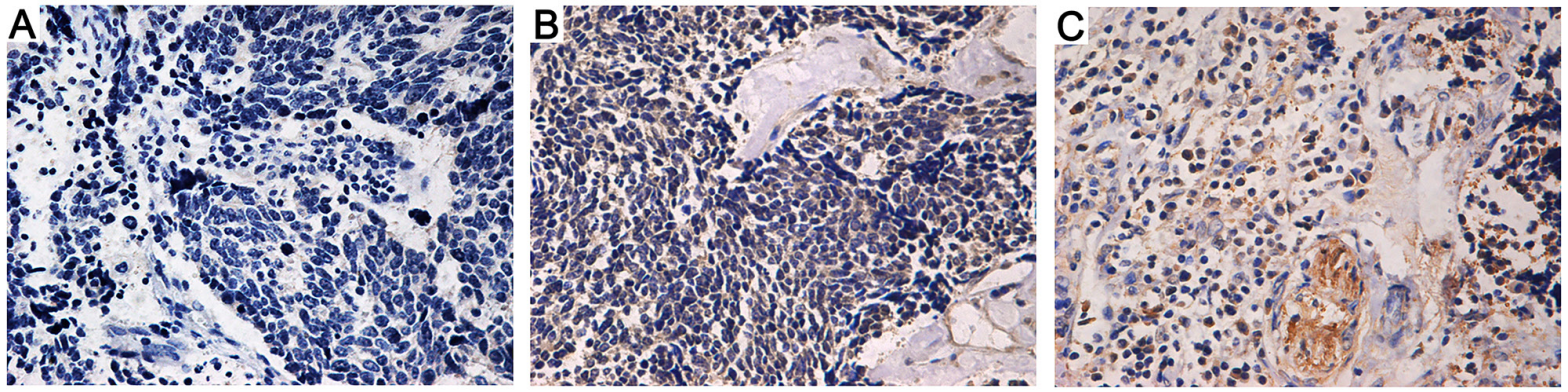
Immunohistochemistry staining of osteopontin (OPN) in lung cancer tissues (×400 magnification) **(A)** Weak expression or focal expression (1+). **(B)** Moderate expression with focal strong expression (2+). **(C)** Strong expression (3+) (·100 magnification).

RNA-sequencing was performed on the obtained human SCLC specimens to determine its gene expression data. Interestingly, 96 mRNAs exhibited significantly different expression levels (P <0.05 and False Discovery Rate (FDR) < 0.5), including 54 upregulated mRNAs and 42 suppressed ones (Figure 1). Of note, OPN was one of the most upregulated mRNAs and few literatures focused on its role in SCLC, so far. Thus, 168 paired paraffin-embedded tissue samples were subjected to qPCR analysis to further confirm the RNA-sequencing findings. As expected, OPN mRNA expression was significantly up-regulated in SCLC tissues compared with adjacent non-neoplastic tissues (Figure 2, P<0.05). In addition, as illustrated in Figure 3, representative cytoplasmic staining of OPN was observed via immunohistochemical analysis. Thirty tumors showed negative expression (-), 48 showed weak or focal expression (1+), 64 showed moderate expression with focal strong expression (2+), and 26 showed strong expression (3+).

### Elevated OPN expression is associated with prognosis in SCLC

**Figure 4:**
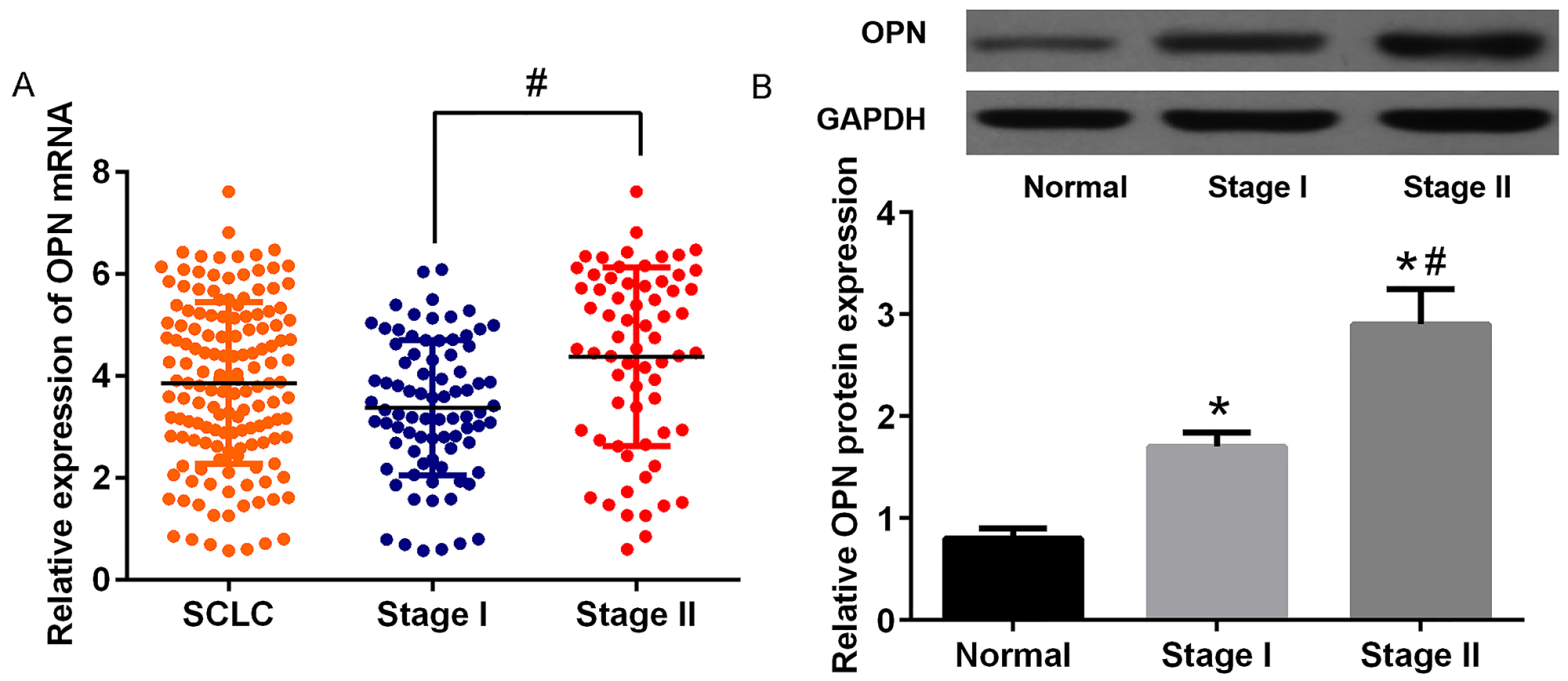
The expression of OPN in different stages of SCLC patients **(A)** Relative expression levels of OPN in 2 different stage of SCLC samples. **(B)** Protein expression of OPN in SCLC tissues samples.*P<0.05 vs. Normal group; #P<0.05 vs. stage I.

**Figure 5:**
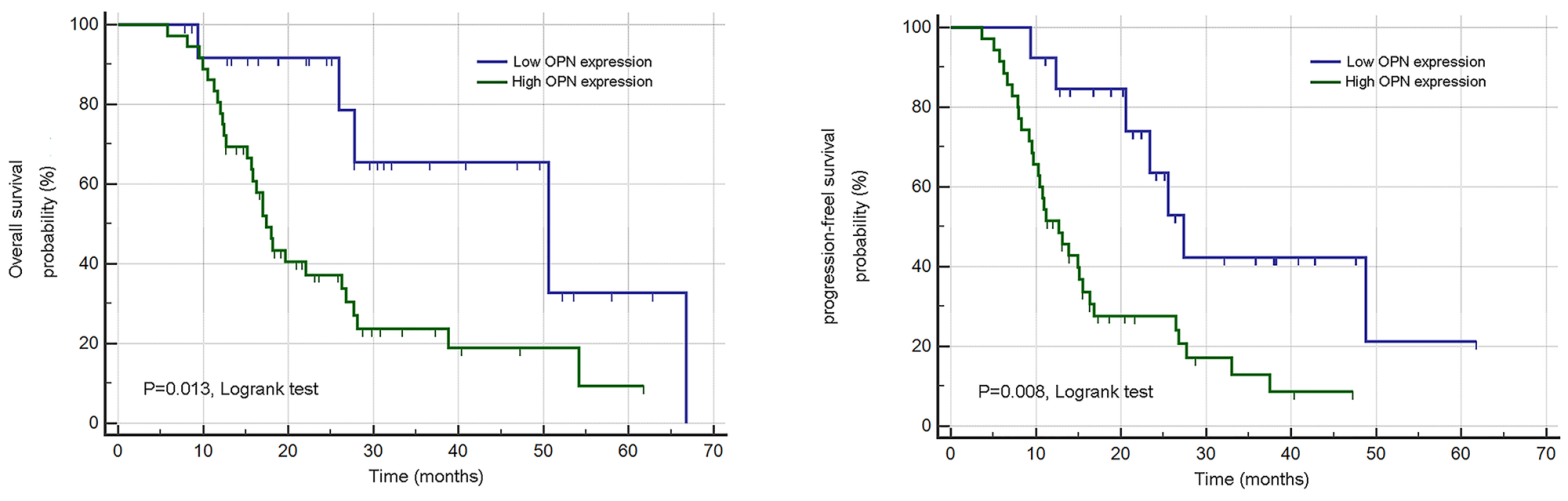
Overall and progression-free survival curves produced using the Kaplan–Meier method by log-rank test for OPN

To determine the clinical significance of OPN in SCLC, we explored the relationship between the OPN expression level and clinical stage of 168 subjects, discovering that increased expression of OPN in stage II patients remained statistically significant relative to stage I patients in this analysis (P<0.05; Figure 4A). In accordance with above finding, OPN protein expression levels were significantly higher in SCLC compared with normal cohorts and levels of OPN were significantly higher in stage II cohorts compared to stage I cohorts (P<0.05; Figure 4B). Above results led to the hypothesize that OPN is potentially associated with the development of SCLC. Thus, the prognostic significance of OPN was investigated. Total patients were assigned to high OPN expression (n=97) and low expression (n=71) group according to the cut-off value. Unexpectedly, the results of Kaplan–Meier survival analysis indicated that high level of OPN expression was correlated with decreased OS (P=0.013) and PFS (P=0.008) (Figure 5). These results strongly indicated that OPN might serve as indicators in SCLC prognosis, which encouraged us to further investigate the correlations between the expression of OPN and clinicopathologic features. The results are shown in Table 1. Abundant OPN was significantly related to pT classification (P=0.001) and pN classification (P=0.017). However, there was no significant association between OPN and other variables, including age, gender and tumor size. Spearman’s correlation analysis showed significantly positive correlations between positive OPN and pathologic tumor classification (pT classification) (r=0.48, P<0.001), as well as pathologic lymph node classification (pN classification) (r=0.54, P<0.001). In addition, to determine independent prognostic factors for overall survival (OS) and progression-free survival (PFS), Cox multivariate analysis was performed. The results revealed that abundant OPN was a strong independent prognostic factor for OS (HR: 4.83 (95% CI: 1.84–11.30), P<0.001) and PFS (HR: 2.87 (95% CI: 1.38–5.73), P<0.001), as well as pT classification and pN classification. Detailed information was provided in Table 2.

**Table 1:** Demographic, clinical characteristics and osteopontin (OPN) expression

Clinical characteristics	Number of cases	OPN expression		*P* value
		Low	High	
Age(years)				
<60	98	40	58	0.771
≥60	70	31	39	
Gender				0.480
Male	115	46	69	
Female	53	25	28	
Tumor size (cm)				0.087
<4	90	44	46	
≥4	78	27	51	
Pathologic tumor classification				0.001
pT1	43	30	12	
pT2	93	30	63	
pT3	32	10	22	
Pathologic lymph node classification				0.017
pN0	101	53	48	
pN1	67	18	49	

**Table 2:** Multivariate Cox proportional hazard model analysis by classification of osteopontin

Characteristics	Overall survival		Progression-free survival	
	HR(95%CI)	*P* value	HR(95%CI)	*P* value
Age(years)				
<60	1.00(reference)			
≥60	2.36(0.77-8.99)	0.425	1.21(0.44-2.39)	0.512
Gender				
Male	1.00(reference)			
Female	1.92(0.54-6.31)	0.287	1.36(0.57-3.83)	0.474
Tumor size (cm)				
<4	1.00(reference)			
≥4	0.96(0.44-2.31)	0.303	0.91(0.61-1.96)	0.451
Pathologic tumor classification				
pT1	1.00(reference)			
pT2-3	1.51(1.20-4.22)	0.007	1.49(1.13-3.24)	0.012
Pathologic lymph node classification				
pN0	1.00(reference)			
pN1	1.38(1.09-2.56)	0.024	1.28(1.02-1.99)	0.037
OPN expression				
low	1.00(reference)			
high	4.83(1.84-11.30)	<0.001	2.87(1.38-5.73)	<0.001

OPN, osteopontin; HR, hazard ratio; CI, confidence interval.

### Role of OPN in epithelial-mesenchymal transition in SCLC

**Figure 6:**
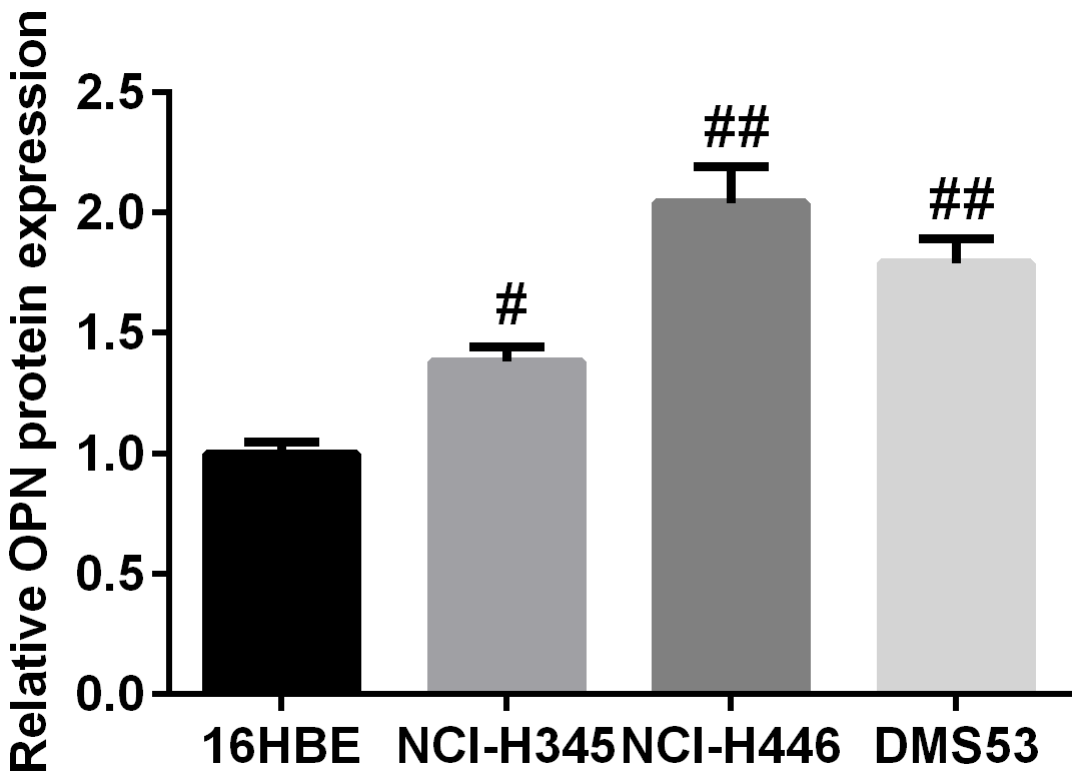
The expression profile of OPN in three SCLC cell lines and human normal bronchial epithelial cell line 16HBE

**Figure 7:**
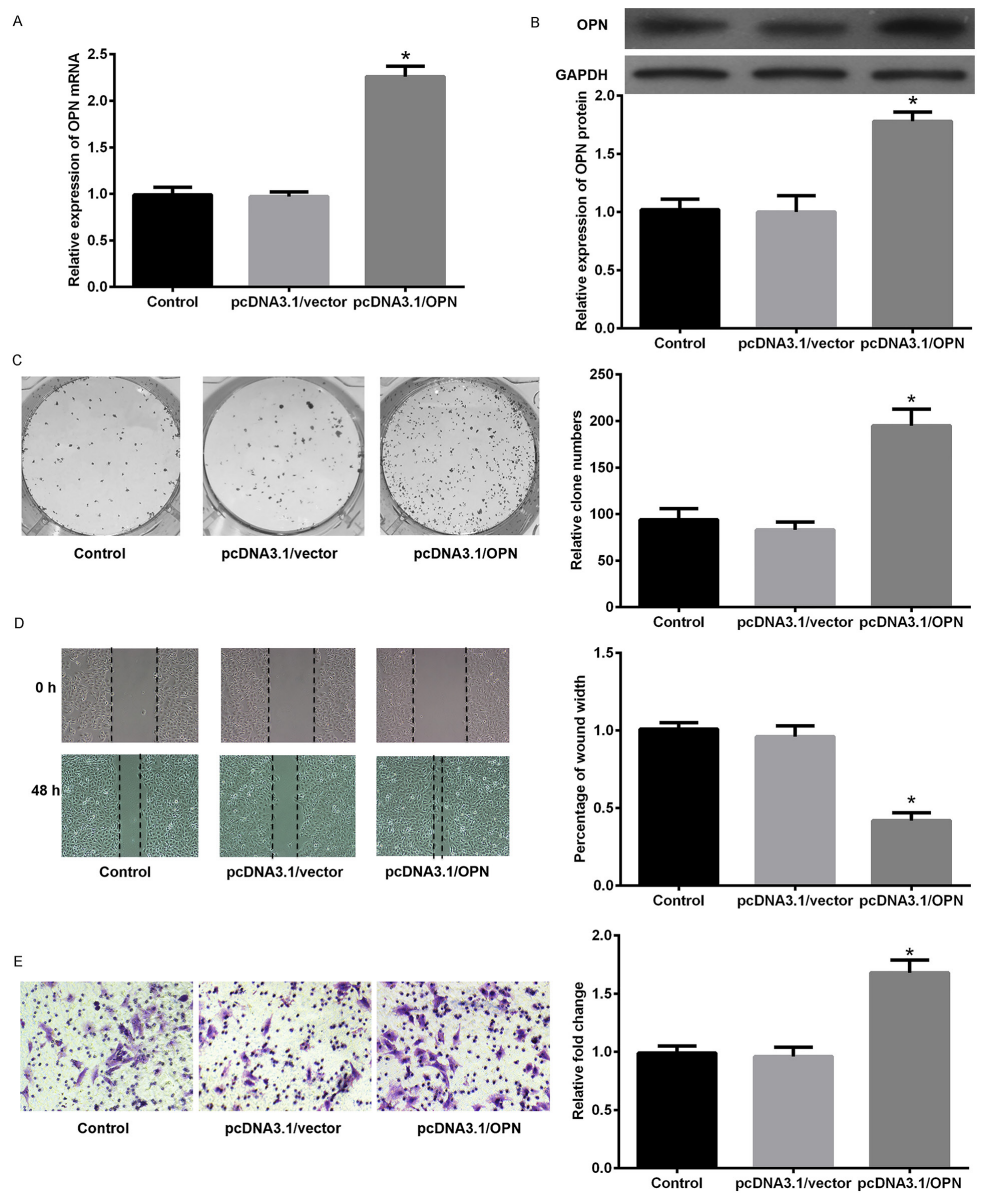
Forced expression of OPN promoted cell proliferation, migration, and invasion ability in SCLC DMS53 cells were transfected with pcDNA3.1/OPN or control vector and the expression of OPN or GAPDH was detected by qPCR **(A)** and western blot **(B). (C)** Clone formation assays revealed differences in the growth of the indicated cell lines. **(D)** Measurement of *in vitro* cell migration by wound healing assays. **(E)** Transwell invasion assays of the indicated cell lines. *P<0.05 vs. NC siRNA.

**Figure 8:**
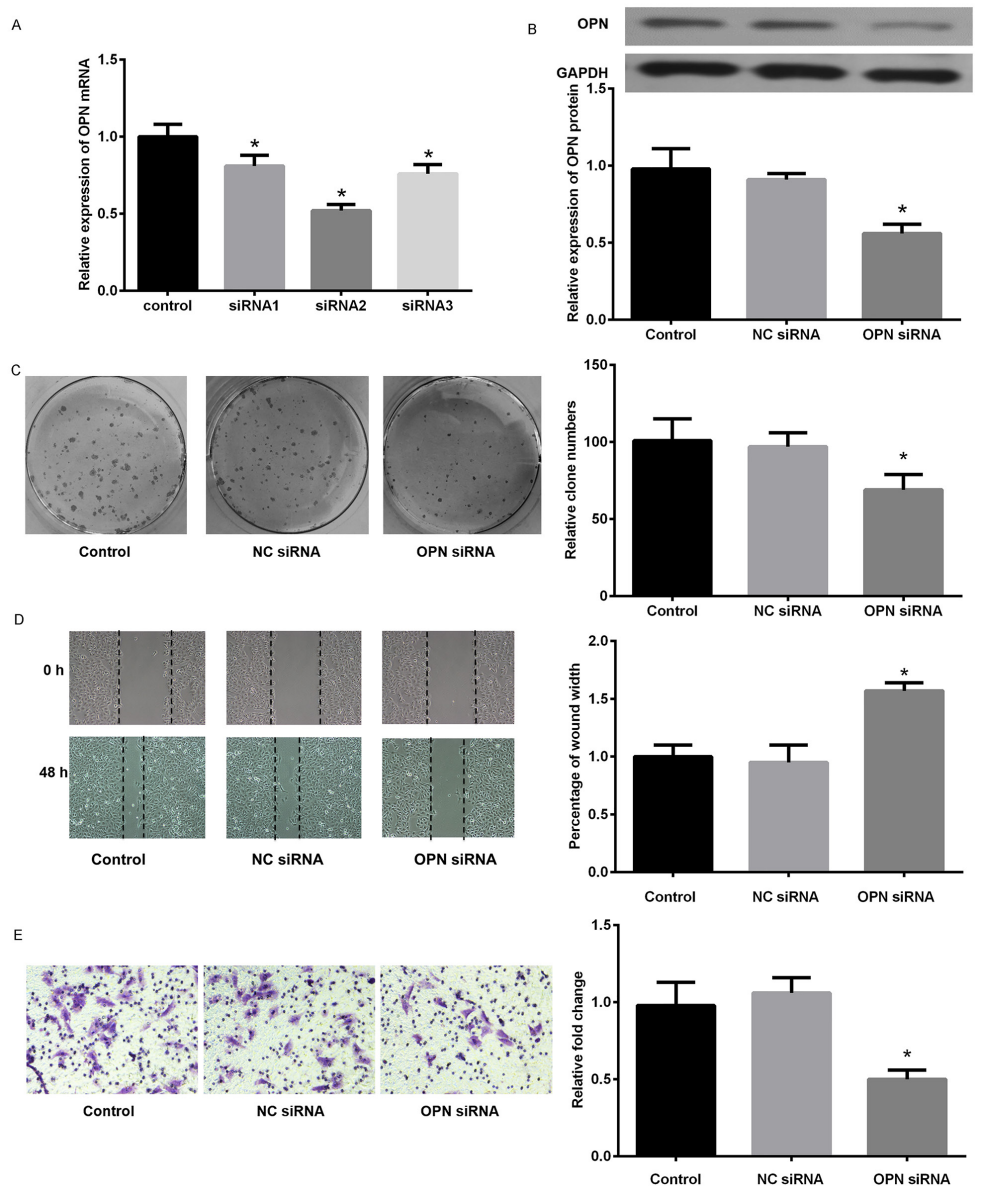
Knockdown of OPN suppressed proliferation, migration, and invasion of small lung cancer cells **(A)** Relative expression of OPN mRNA after interference with three different OPN siRNAs. **(B)** Expression of OPN protein in the control group, negative control siRNA group (NC siRNA), and OPN siRNA group (siRNA2). **(C)** Effects of OPN knockdown on colony formation of NCI-H446 cells transfected with OPN or NC siRNA. **(D)** Wound healing assay on NCI-H446 cells transduced with OPN or NC siRNA. **(E)** The effect of OPN on invasion of NCI-H446 cells was assessed by a Transwell assay. *P<0.05 vs. NC siRNA.

**Figure 9:**
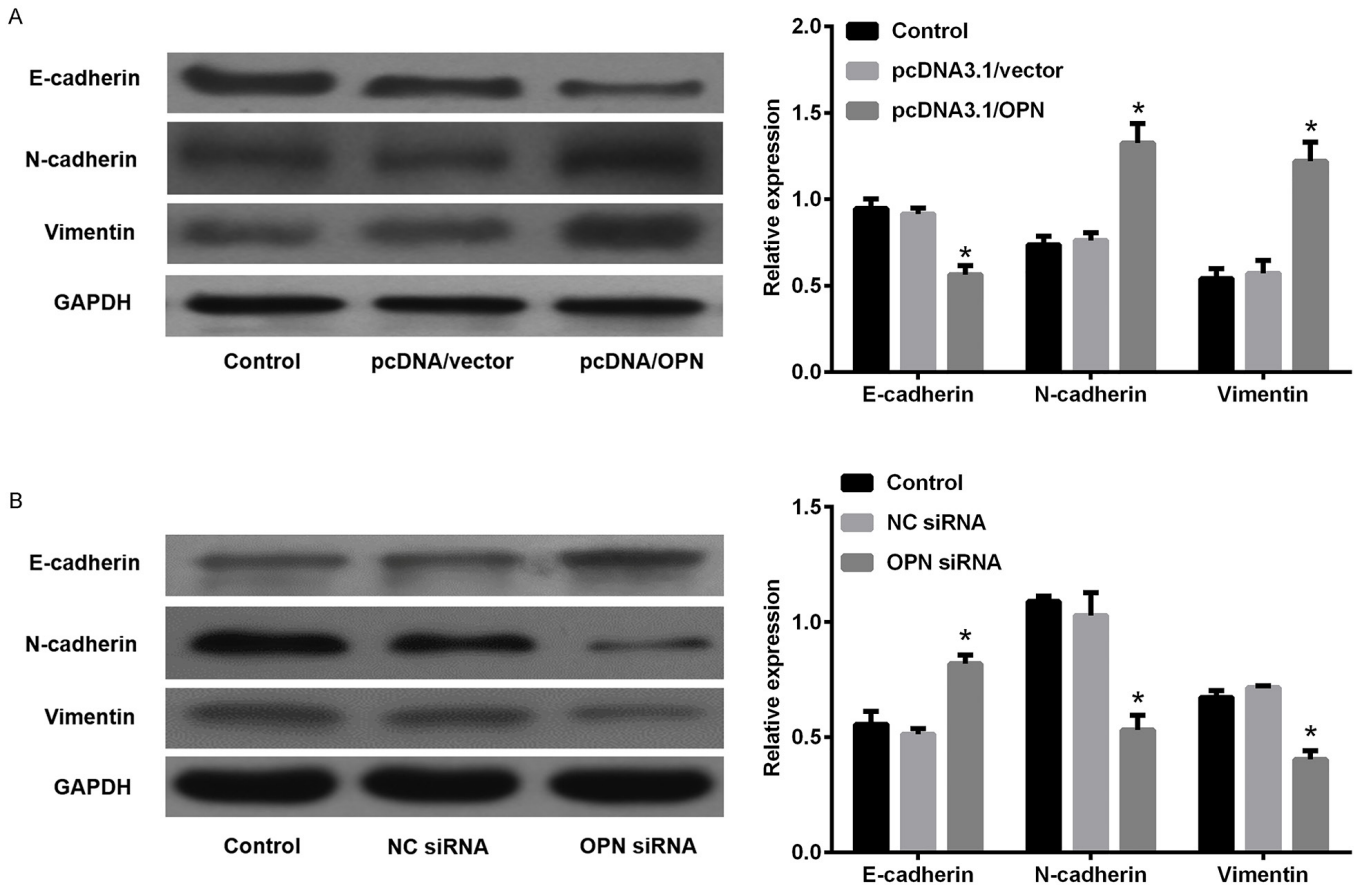
Effects of osteopontin (OPN) on the expression of epithelial-to-mesenchymal transition (EMT) in small cell lung cancer cells **(A)** DMS53 cells were transfected with pcDNA3.1/OPN or control vector and western blot analysis was used to study changes in protein expression of Vimentin, N-cadherin, and E-cadherin. **(B)** Cells were transfected with OPN or NC siRNA and EMT markers were then detected by western blot analysis. *P<0.05 vs. NC siRNA.

Since the findings above, *in vitro* experiments were performed to study the biological function of OPN in EMT in SCLC cells. As illustrated in Figure 6, OPN expression level was markedly increased in SCLC cell lines compared with 16HBE detected by qPCR. Subsequently, the amount of OPN in DMS53 cells after transfection with pcDNA3.1/OPN or corresponding control vector was determined. Figure 7A, 7B showed the level of both OPN mRNA and protein were significantly higher in pcDNA3.1/OPN group than in control groups (all P<0.05). The effects of OPN on proliferation, migration, and invasion of SCLC cells were investigated. Cell proliferative ability was significantly promoted by OPN overexpression, as detected by colony formation assays (Figure 7C). The results of wound healing and Transwell assay indicated that cell migration and invasion ability was increased when OPN was upregulated (Figure 7D, P<0.05). To further explore whether konckdown of OPN could achieve the suppression of cell proliferation, migration and invasion ability, NC siRNA or OPN siRNA was used. Figure 8A shows the capacities of the three synthetic siRNAs to knock down OPN; siRNA2 showed the best performance (P<0.05) and was used in all following experiments. The expression of OPN protein was evidently reduced in the OPN group compared to the NC siRNA and control groups (P<0.05, Figure 8B). As a result, we found that OPN knocking-down inhibited cell proliferation, migration and invasion (Figure 8C-8E). EMT usually causes pathological migration and invasion during cancer progression. Consequently, western blotting was carried out to investigate the effects of OPN on EMT in DMS53 cells. OPN overexpression increased expression of the mesenchymal markers N-cadherin and vimentin and decreased the expression of the epithelial marker E-cadherin, while knock down of OPN exerted the opposite effects on EMT markers (Figure 9A, 9B). Taken together, these data indicate that OPN affects the proliferation, migration, and invasion of SCLC cells and is involved in EMT in SCLC.

## DISCUSSION

In the present study, we firstly identified that OPN were frequently upregulated in SCLC through RNA-sequencing and validated by qPCR, immunohistochemical and Western blot. We then analyzed its relationship with clinical and prognostic characteristics and found that OPN expression levels were strongly associated with poor prognosis. Further studies found that OPN was an independent prognostic factor for OS and PFS. Moreover, subsequent *in vitro* studies indicated that OPN affected cell proliferation, migration, and invasion and promoted EMT in SCLC. Our results for the first time suggested that abundant OPN might be useful for predicting the prognosis in SCLC.

Emerging evidence has shown that aberrant OPN expression is associated with tumorigenesis and metastasis in kinds of tumors [15, 16]. Researchers have reported the important role of OPN in neoplastic transformation and cancer progression in breast cancer, and has the ability to induce breast cancer cells to metastasize to the bone marrow [17]. In addition, high levels of OPN indicate a poor outcome in terms of OS (HR=2.22, 95% CI: 1.23–4.00), which suggested that OPN could be considered as a prognostic marker for breast cancers [18]. Another study suggests that OPN positivity is a useful biomarker for predicting the risk of hematogenous metastasis in gastric cancer patients [19]. These studies consistently implied that OPN overexpression could serve as a poor prognostic biomarker for cancer patients. Moreover, a meta-analysis documented that increased expression of OPN protein may be significantly associated with poor prognosis in patients with NSCLC [8, 20]. And interestingly, the plasma OPN levels are significantly increased after definitive radiotherapy and correlate with survival and outcome of non-SCLC [21]. However, we didn’t investigate the plasma OPN expression levels in SCLC patients in current study. Importantly, one study indicated that OPN enhances chemo-resistance to cisplatin in human SCLC cells and inhibits cell apoptosis, which could explain the poor prognosis of cancer patients with high OPN expression [9]. In line with previous studies, our study indicated that OPN positivity was associated with poor overall survival in SCLC. Subsequent multivariate analysis found that abundant OPN was a reliable independent prognostic factor for OS (HR=4.83) and PFS (HR=2.77). Activation of EMT is associated with dissolution of intercellular junctions through the internalization and down-regulation of epithelial markers such as E-cadherin, and up-regulation of mesenchymal markers, including N-cadherin and vimentin [22]. EMT is considered as a reversible process that often occurs at the invasive front of many metastatic cancers [23, 24]. In this study, we for the first time found that OPN overexpression could promote, whereas, knockdown could inhibit cell proliferation, migration, and invasion and that an absence of OPN could prevent EMT, uncovering the underlying mechanism for the role of OPN in SCLC. A recent study suggested that OPN regulates EMT through protein kinase B (PKB/AKT) and Extracellular regulated protein kinases (ERK1/2) signaling pathway [25]. And OPN is able to active PI3K-AKT-Twist pathway leading to EMT [26]. In addition, OPN was reported to influence other EMT pathways including ZEB1/2 [27] and Snail [28]. However, how OPN is involved in the cell metastasis still needs to be fully elucidated.

Although we have assessed the value of OPN in SCLC, some limitations of our work should not be ignored. We explored the prognostic value of OPN in a relatively small cohort and the conclusion was obtained only in limited-stage patients because tissues samples could hardly obtained from extensive-stage patients. as noted, we investigated the changes in the levels of only EMT markers in this study, the mechanism by which OPN regulates EMT is still unanswered. More results with a large cohort of SCLC patients are quite needed.

In summary, we suggest that OPN show promise as markers for decreased overall survival in SCLC, was a reliable prognostic factor for SCLC. Furthermore, OPN promotes cancer proliferation, migration and stimulates the EMT and may be a promising therapeutic target for the treatment of SCLC. Specific silence of OPN could be a future direction to develop a novel therapeutic strategy for SCLC patients.

## MATERIALS AND METHODS

### Subjects

In this work, 168 pairs of tumor tissues and paired corresponding normal tissues were obtained from patients who underwent radical resection without either chemotherapy or radiotherapy before operation at the Zhongshan Hospital Affiliated to Fudan University and Dongfang Hospital Affiliated to Tongji University from January 2010 to October 2014. All patients were pathologically diagnosed as limited-stage SCLC and defined as stage I and II with a combined approach [29]. The clinical characteristics of all subjects were recorded. The patients were followed up until October 2015, with a median follow-up period of 49 months (range from 5.8–66.7 months). Among the 168 patients, 115 were male and 53 were female, with a median age of 56 years (33–82 years). During the hospitalization, 71% (119/168) of the patients received totally adjuvant treatment before surgery; 32 patients received chemotherapy and radiotherapy, 11 patients received chemotherapy only, and 6 patients received radiotherapy only after surgery. Overall survival (OS) was defined as the time from surgery to death for any reason. Progression-free survival (PFS) was measured from the start of platinum-based therapy to tumor progression, as determined by the date of the imaging study at which the radiologist’s report noted progression. The ethics committee of the Zhongshan Hospital Affiliated to Fudan University approved this research. All participants provided written informed consent.

### RNA sequencing

Total RNA was extracted from paired tissues and cells by using TRIzol reagent (Invitrogen, Carlsbad, CA) strictly according to the manufacturer’s protocol. The quality of RNA was analyzed using a 2100 Bioanalyzer system (Agilent Technologies). The cDNA libraries were prepared by using mRNA-Seq Sample Prep Kit (Illumina, San Diego, CA, USA). RNA sequencing was performed using Illumina Genome Analyzer using the standard protocol. RNA-seq data was normalized in R v3.2.3 statistical environment (http://www.r-project.org) using DESeq Bioconductor library using a negative binomial distribution [30].

### Quantitative-polymerase chain reaction (qPCR)

To validate the data of RNA sequencing, cDNA was synthesized from 2 μg total RNA extracted from 168 paired tumor samples and the quantification of mRNA was performed using a Primescript™ RT reagent kit (TakaRa, Dalian, China) following the manufacturer’s instructions. qPCR was performed on an ABI 7300 RT-PCR Detection System (Applied Biosystems, Foster, CA, USA) using a standard SYBR Green II PCR kit (TakaRa). Primers for OPN (forward, 5’- CTCCATTGACTCGAACGACTC-3’and reverse, 5’- CAGGTCTGCGAAACTTCTTAGAT-3’) were purchased from Ribobio. qPCR reactions were performed based on the following: 95°C for 2 min; 40 cycles at 95°C for 10 s; 60°C for 30 s; and 72°C for 15 s. GAPDH (forward, 5’-GCACCGTCAAGGCTGAGAAC-3’ and reverse, 5’-TGGTGAAGACGCCAGTGGA-3’) mRNA were used to normalize the target genes via the 2-ΔΔCt method. All reactions were performed in triplicate.

### Immunohistochemical analysis

Immunohistochemical analysis was then conducted for OPN detection. Resected lung tissues were fixed in 10% formaldehyde and embedded in paraffin. Sections were cut into 4-μm thick slices and all sections were routinely deparaffinized and rehydrated followed by washing with 0.01 M phosphate-buffered saline (pH=7) three times. Antigen retrieval was performed by placing the sections in 0.01 M sodium citrate buffer and exposure to two rounds of microwave heating of 10 min at 500 W. After being rinsed in PBS and 3% H2O2, the sections were incubated with normal goat serum at 37°C for 30 min. Following incubation with anti-OPN polyclonal antibody (1:100, Proteintech Group, USA) overnight at 4°C, sections were incubated with biotinylated secondary antibodies and rinsed again with PBS. After incubation with streptavidin-HRP for 30 min at room temperature, the sections were rinsed in PBS, visualized by reaction with 3,3′ diaminobenzidine (DAB) (Solarbio, Beijing, China), and then nuclear counterstained with hematoxylin. Finally, they were dehydrated, made transparent, covered with coverslips, and sealed with neutral gum. The results were evaluated in five areas of each slide, and OPN expression was classified as negative (-); weak or focal expression (+); moderate expression with focal strong expression (2+); and strong expression (3+).

### Cell culture

The human SCLC cell lines NCI-H345, NCI-H446 and DMS53, as well as the normal bronchial epithelial cell line 16HBE, were purchased from the American Type Culture Collection (Manassas, VA) and maintained at 37°C in RPMI 1640 containing 10% fetal bovine serum (Sigma-Aldrich, St. Louis, MO, USA), 1% sodium pyruvate, 25 mmol/L HEPES, 4.5 g/L glucose, and 1% of 100 U/mL penicillin and streptomycin.

### Vectors and cell transfection

To construct the OPN expression vector, the OPN gene (forward, 5′-GATATAGATACTGATAGATCTAGATATG-3′ and reverse, 5′-CTCTCTTGTCTAGTCTGTATGTTC-3′) was amplified via PCR and cloned into pcDNA3.1 using Life Technologies (Guangzhou RiboBio Co., Ltd., Guangzhou, China). Sequencing was performed to verify the clones; the positive clone was designated pcDNA/OPN. SiRNAs targeting OPN at three different loci were synthesized by Guangzhou RiboBio Co., Ltd. and confirmed by enzyme digestion analysis. The OPN siRNA2 sequence was: 5′-ACGAUGUCAUCCUAAGACCACUUGU-3′ and the NC siRNA sequence was: 5′-CUGCCUACACAUUCCGAUGUUGGAA-3′. DMS53 cells were cultured in 6-well plates with the density of 1 × 10^5^ cells/volume. Transfections were performed with Lipofectamine 2000 reagent (Invitrogen, Carlsbad, CA)) as described by the manufacturer.

### Western blot

Total protein was extracted from cells using 1% RIPA Lysis Buffer (Beyotime, Jiangsu, China) and quantified with the BCA protein assay kit (Beyotime). Then equal amounts of protein were separated on 10% sodium dodecyl sulfate polyacrylamide gel electrophoresis (SDS-PAGE) and transferred to PVDF membranes (Millipore, Bedford, MA). The membrane was blocked with Tris buffer containing 5% non-fat milk in TBS-T at 4°C followed by incubated with primary antibodies including anti-OPN (1:1000, Abcam), anti-E-cadherin (1:500, Abcam), anti-N-cadherin (1 μg/ml, Abcam), anti-Vimentin (1:2000, Abcam), and anti-GAPDH (1:1000, Cell Signaling Technology, Massachusetts, USA) overnight at 4°C. And then secondary antibodies (Goat anti-rabbit or anti-mouse IgG H&L) (1:5000, Santa Cruz, USA) was incubated for 1 h at room temperature. Results were detected using an ECL detection system (Millipore) according to the manufacturer’s instructions.

### Colony formation assays

First, pcDNA/vector or pcDNA/OPN, OPN siRNA- or NC siRNA were transfected in DMS53 cells for 48 h. Cell proliferation was monitored using a colony formation assay. Then, 3000 cells were grown in 6-well plates and maintained in DMEM containing 10% FBS. After 14 days, cells were fixed with 4% paraformaldehyde for 15 min and stained with 0.1% crystal violet (Beyotime, Jiangsu, China) for 5 min. Visible colonies were counted manually.

### Cell migration and invasion assays

For the wound-healing assay, cells were seeded into six-well plates, cultured overnight, and transfected with relevant vectors. Upon reaching appropriate confluence, a sterilized pipette tip was used to scratch a fine line with the same width on the cell layer, followed by washing twice with 500 μl phosphate-buffered saline. After culturing for an additional 48 h in serum-free medium, Photos were taken at 0, 12 and 24 h after the scratching. For the migration assays, cells at a density of 8x10^4^ cells/well were cultured 24-wells that had been pre-coated with Matrigel (R&D Systems, Minneapolis, MN, USA) were used. Serum-free media was placed in the upper chamber of the insert (8-μm pore size, BD, USA), while medium containing 10% FBS in the lower compartment. After 48 h of incubation, cells that remained in the upper chamber were removed with cotton wool and those that invaded into the lower surface were fixed with 4 % paraformaldehyde and stained with 0.1% crystal violet. Photos were taken at×100 magnification. Each experiment was repeated three times.

### Statistical analyses

Data are shown as the median ± standard deviation of three independent experiments. SPSS 19.0 software (SPSS Inc., Chicago, IL, USA) was used for statistical analysis. Differences between groups were evaluated by Pearson’s chi-square test. Spearman’s rank correlation analysis was used for ranked data. OS and PFS were determined using the Kaplan–Meier methods and differences were analyzed via log-rank test. Multivariate analysis was performed using Cox’s proportional hazard model. Values of P<0.05 were considered statistically significant.
